# Binge eating, purging and non-purging compensatory behaviours decrease from adolescence to adulthood: A population-based, longitudinal study

**DOI:** 10.1186/1471-2458-12-32

**Published:** 2012-01-13

**Authors:** Dawit Shawel Abebe, Lars Lien, Leila Torgersen, Tilmann von Soest

**Affiliations:** 1 Norwegian Social Research (NOVA), P.O. Box 3223, Elisenberg 0208 Oslo, Norway; 2 Division of Mental Health, Norwegian Institute of Public Health, Oslo, Norway; 3 Division of Mental Health and Addiction, University of Oslo, Oslo, Norway

## Abstract

**Background:**

Subclinical forms of eating disorders (ED) are highly prevalent, but relatively little is known about age trends, gender differences and distinctions among symptoms. This study investigates age trends and gender difference in binge eating, purging and non-purging compensatory behaviours (CB) and the relationship of such behaviours to psychosocial problems.

**Methods:**

Data from the national representative longitudinal study "Young in Norway" (ages 14-34 years) were analysed using *χ*^2 ^tests, logistic random intercept models and analyses of covariance.

**Results:**

For both genders, a decrease was found in the prevalence of CB from age 14-16 years to 23 years and over. For binging, however, a significant decrease was found only for females, whose binge eating also declined more markedly over time than did males'. A significant gender difference was detected for purging, with females at higher risk. Purging was related to particularly serious symptoms of psychosocial problems: Those who purged had significantly higher levels of appearance dissatisfaction, anxiety and depressive symptoms, alcohol consumption, self-concept instability and loneliness than those with symptoms of other forms of disordered eating.

**Conclusions:**

Individuals affected by purging need to be targeted as a high-risk group. The distinction in severity among the subclinical ED may indicate the need for the reformulation of the eating disorder not otherwise specified category in the Diagnostic and Statistical Manual of Mental Disorders-V.

## Background

Binge eating and compensatory behaviours (CB) are the most frequent symptoms of full- and sub-threshold forms of eating disorders (ED) [1,2]. CB are inappropriate weight control behaviours and are divided into purging behaviours such as self-induced vomiting and the use of laxatives and diuretics, and non-purging behaviours such as the use of diet pills, excessive exercise and dietary restraint. These behaviours, with or without binge eating, are the essential behavioural criteria for the current classifications of ED and represent the central features of bulimia and eating disorders not otherwise specified (EDNOS) [2]. However, current classification systems for ED have been criticised for assigning too large a proportion of diagnosed ED patients to the residual EDNOS category [3] - nearly half of those receiving treatment [4]. Recent studies argue that binge eating with and without purging and non-purging CB needs to be more appropriately classified within diagnostic systems of ED [5-9]. Some suggest that purging without other ED symptoms (purging disorder) should be included in future classification schemes [5,10,11]. In contrast, others propose that future diagnostic categories be reserved for combinations of binge eating with CB, whether purging or non-purging [7,9].

In addition to clinical data, population-based studies examining the epidemiology of subclinical symptoms of binge eating and CB may prove informative for the future classification of ED. Although a few such studies have been published [5-9], they provide limited information concerning the prevalence of symptoms and correlates with other psychosocial variables, and also whether such symptoms have different epidemiological patterns and co-morbidity. Thus, by using data from a large population-based longitudinal cohort study in Norway, this study aims to investigate the epidemiology of binge eating and purging and non-purging CB. It focuses particularly on age trends and gender differences in reported symptoms and the relationship of the symptoms to psychosocial problems.

Concerning age trends, few prospective community-based studies to date have examined changes in binge eating and CB from adolescence to adulthood [12-14]. Heartherton and colleagues suggest that the transition to adulthood is related to a decline in binge eating and CB for women [12]. Recently, however, another study reported no significant decreases in point-of-time prevalence of purging among men and women over time, indicating stability in the frequency of purging behaviours across cohorts and time [5]. These studies did not explore age-related changes, and such exploration is vital if we are to gain an improved understanding about the progress of binge eating and CB from adolescence to young adulthood.

Unlike the full-threshold forms of ED, which have disproportionately high rates among females, binge eating and CB are found at comparatively high rates in both genders [15]. However, there remains a female preponderance for purging behaviours [16], whereas males and females are equally apt to report binge eating and excessive exercise [15]. Such findings from community-based studies provide ample support to capture the types of binge eating and CB specific to each gender.

So far, few studies have examined how specific subclinical forms of ED such as binging, binging combined with CB, purging and non-purging are related to measures of general psychopathology. One exception is Mond and colleagues, who reported that individuals who engaged in a combination of recurrent binge eating and CB, whether purging or non-purging, had significantly higher levels of dietary restraint, eating and weight concerns, general psychological distress and functional impairments than those engaging in CB alone [7]. Moreover, Haedt and Keel showed that purging behaviours per se can demonstrate distinctive psychopathology; individuals with purging behaviour reported significantly greater perfectionism and more impairment in social relationships and educational satisfaction than a non-ED group [5]. Further research on characteristics of such eating problems has been strongly recommended [9,10]. To validate whether binge eaters with or without CB are discriminable, it is important to show that these groups have significant and distinctive patterns of psychopathology.

In conclusion, more knowledge about the epidemiology of binge eating and CB is needed. This population-based study aims to examine this issue by looking at age trends and gender differences in the prevalence of such symptoms. Moreover, it investigates how specific symptoms of binge eating and CB are related to a comprehensive range of psychosocial variables. These variables include measures of general eating problems and other psychosocial problems such as appearance satisfaction, anxiety and depressive symptoms, self-worth, alcohol consumption, relationship to parents, self-concept, social support and loneliness.

## Methods

### Procedure and participants

We analysed data from the longitudinal study Young in Norway [17,18], a national representative study that was conducted at four time points: 1992 (T0), 1994 (T1), 1999 (T2) and 2005 (T3). The initial sample at T0 comprised 12 287 students in grades 7-12 (12-20 years of age) from 67 representative schools in Norway. Every school in the country was included in the register from which the schools were selected, and each grade was equally represented. The sample was stratified according to geographical region and school size, which in Norway is closely related to the degree of urbanization. Each school's sampling probability was proportional to the number of students in the school, giving each student an equal probability of being selected. Detailed information about the sampling procedure is presented elsewhere [17,18]. The response rate at T0 was 97%.

Three of the participating schools at T0 were not part of the 1994 follow-up study (T1; 14-22 years of age). At another school, a burglary in the school's archives resulted in the loss of the project's identification records. In all, then, 9679 students from 63 schools were eligible to complete the T1 questionnaire. Because a considerable proportion of the students had completed their 3-year track at the junior or senior high school they were attending at T0, those no longer at the same school at T1 received the questionnaire by mail. For this group the response rate was 68%; for those at their original schools it was 92%.

Only students who completed the questionnaire in school at T1 (n = 3844) were followed up at T2 due to the comparatively lower response rate from those who received the T1 questionnaire by mail. Because the survey was originally planned as a 2-wave study, informed consent had to be obtained again at T1. Of the total number of consenting individuals at T1 (n = 3507, 91%), 2923 (84%) responded to the questionnaire they received by mail at T2 (19-28 years of age). The overall participation rate at T2, based on all eligible students at T0 who still were at their original school at T1, was therefore 68%.

In 2005 (T3), all those who had consented at T1 to the follow-up were again invited to participate (25-34 years of age). In all, 2890 of the 3507 T1 participants (83%) completed the T3 questionnaire, resulting in an overall response rate of 67%. For purposes of this study, data from T1-T3 were used, as binge eating and CB were not measured at T0.

### Measures

**Binging and compensatory behaviours **were measured by the six items on the severity scale of the Bulimic Investigatory Test, Edinburgh (BITE) [19]. These items measure the frequency of binge eating and CB, ranging from 'never' to '2-3 times per day.' The scale consists of the following questions: 'Do you ever binge on large amounts of food?'; 'Do you ever fast a whole day?'; 'Do you take diet pills to help you lose weight?'; 'Do you take laxatives to help you lose weight?'; 'Do you take diuretics to help you lose weight?'; 'Do you make yourself vomit to help you lose weight?'; and 'Do you train very hard to help you lose weight?'. For estimating prevalence, we set the minimum frequency of each behaviour to once per week [5] and subsequently dichotomised the frequencies into less than once per week (0) and once per week or more (1). Next, we created four groups based on patterns of binge eating and CB. The four groups were: binging without CB; binging combined with any form of CB (binging-CB); purging CB (self-induced vomiting, taking laxatives, or taking diuretics); and non-purging CB (taking diet pills, fasting or exercising excessively). Individuals with binge eating symptoms were excluded from the purging CB and non-purging CB groups, thereby ensuring that those groups consisted of individuals reporting only category-specific symptoms. All groups were mutually exclusive. Due to the small sample size of the purging CB group, we could not separate binge eating with CB into binge eating with purging CB and binge eating with non-purging CB.

**Eating problems **were also assessed by two other general measures. The first, the BITE scale, consists of 30 items measuring a broad range of symptoms as well as attitudinal and cognitive aspects of bulimia [19]. All items were rated on a scale ranging from 1 to 4, and a mean score was calculated, with high scores indicating high levels of symptoms. The second additional measure, the Eating Attitude Test-12 (EAT-12), measures eating problems and concerns related to dieting, bulimia and food preoccupation, and oral control [20,21]. The scale has 12 items with response alternatives ranging from 1 ('never') to 4 ('always'). Mean scores were calculated, with high scores reflecting high levels of eating problems.

**Appearance satisfaction **was assessed by the Body Areas Satisfaction Scale (BASS) [22]. The scale rates respondents' level of satisfaction with seven body areas: face, lower torso, mid-torso, upper torso, muscle tone, weight and height. Response options varied from 1 ('very dissatisfied') to 5 ('very satisfied'). A mean score was computed, with high scores indicating a high level of satisfaction.

**Depressive symptoms **were measured by the 6-item Depressive Mood Inventory constructed by Kandel and Davies [23]. Using a response scale ranging from 1 to 4, participants were asked to restrict their ratings to the preceding week. Mean scores were calculated, with high scores indicating high levels of depressive symptoms.

**Symptoms of anxiety **were measured by six items derived from the Hopkins Symptom Checklist [24]. Item responses ranged from 1 to 4 and were restricted to the preceding week. A mean score was computed, with high scores showing strong symptoms of anxiety [25].

**General self-worth **was measured using the Global Self-Worth subscale of a revised version of the Harter's Perception Profile for Adolescents [26,27]. A 4-point response scale was applied, ranging from 1 ('corresponds very poorly') to 4 ('corresponds very well'). A mean score was computed, with high scores reflecting high self-worth.

**Alcohol consumption **was measured by asking participants to indicate how often they had 'drunk so much that you felt clearly intoxicated' during the preceding 12 months. The response scale ranged from 1 ('never') to 6 ('more than 50 times').

**Relationship to parents **was assessed by a short version of the Parental Bonding Instrument [28]. The scale measures the emotional relationship between participants and parents by focusing on two dimensions, parental care and parental overprotection. Each dimension consists of five items and has a response scale ranging from 1 ('very like') to 4 ('very unlike'). High scores on the care subscale indicate a parent-child relationship based on emotional warmth, closeness and empathy, whereas high scores on the overprotection subscale suggest parental obstruction of independent behaviour, parental control and parental intrusion [28].

**Self-concept **was measured by a revised version of Rosenberg's Stability of Self Scale [29]. The scale has four items, each with a response range from 1 to 4. Low mean scores indicate stability and high scores instability in terms of self-concept [29,30].

**Social support **was measured by five items of the Social Support Questionnaire, modeled after Sarason and colleagues' scale [31]. The response alternatives range from 1 ('very poorly satisfied') to 4 ('very satisfied'). High mean scores indicate respondents' high level of satisfaction with their social support network.

**Loneliness **was measured by a 5-item version of the UCLA Loneliness Scale, each item having response options ranging from 1 ('never') to 4 ('often') [32]. A higher mean score reflects greater loneliness.

**Body mass index **(BMI, kg/m2) was computed from self-reported measures of height and weight. Self-reported BMI has been demonstrated to be a valid measure of actual BMI [33]. **Age **was recorded at the time of each survey. **Gender **was coded as 1 for male and 2 for female.

### Statistical analysis

We applied *χ*^2 ^tests to determine the significance of the differences in prevalence of binge eating and CB at each study time and between males and females. We also carried out *χ*^2 ^tests for trends to assess changes in odd ratios (OR) over time and age. A logistic random intercept model was applied to investigate age- and gender-related changes in binge eating and CB, and analyses of covariance (ANCOVA) were performed to compare groups on continuous measures of general eating and psychosocial problems, controlling for age and gender as covariates. The Tukey-Kramer test was applied for multiple *post hoc *comparisons of groups. Statistical analysis of the data was carried out using Stata SE/11 for Windows.

### Attrition analysis

Since a large proportion of the sample did not respond to the questionnaires at T2 and T3, analyses were conducted to explore the potential impact of certain variables on attrition. Specifically, we performed univariate logistic regression to investigate whether binge eating and/or CB at T1 predicted drop out at T2 or T3. Results of these analyses showed, with one exception, no significant differences in proportions of binge eating and all types of CB between those who dropped out and those who stayed in the study (*p *> 0.05). The exception was that participants who exercised excessively at T1 had a lower risk of drop out at T2 and T3 (OR = 0.76, 95% CI = 0.66-0.87, *p *< 0.01 at T2 and OR = 0.75, 95% CI = 0.65-0.85, *p *< 0.01 at T3).

### Ethical clearance

The study was approved by the Norwegian Data Inspectorate and the Regional Committee for Medical Health Research Ethics. Principles governing biomedical research in humans as stated in the Declaration of Helsinki were followed.

## Results

The mean ages of participants were 17.2, 21.9 and 28.3 years at T1, T2 and T3, respectively. Gender-specific prevalence for binge eating and all forms of CB across all three time points are summarized in Table 1. We found statistically significant trends for binge eating and fasting among males; the prevalence of these behaviours decreased from T1 to T3. For females, the prevalence of binge eating, excessive exercise and fasting decreased significantly from T1 to T3. For both genders, however, the use of diet pills increased significantly over time. Females reported a significantly higher prevalence than males of binge eating and self-induced vomiting at T1, use of diet pills at T2 and T3 and excessive exercise at T1 and T3. In contrast, we found no significant gender difference in the taking of laxatives and diuretics and fasting at each study time. Nonetheless, it is important to note that some of groups have a small sample (n).

**Table 1:** Prevalence of binge eating and compensatory behaviours by time points and gender1

	T1		T2		T3		*χ*^2 ^for time trend (d.f. 1)
		
	N	%	n	%	n	%	
**Males**	3771	46.9	1207	44	1194	43.8	-
Binge eating^a^	368	13.8	79	7.9	56	6.1	49.9***
CB - purging:							
Self-induced vomiting^a^	8	0.2	0	0.0	2	0.2	0.5^ns^
Taking laxatives	9	0.2	1	0.1	0	0	3.7^ns^
Taking diuretics	8	0.2	2	0.2	4	0.3	0.4^ns^
CB - non-purging:							
Taking diet pills^b, c^	12	0.3	3	0.2	10	0.8	4.6*
Excessive exercise^a, c^	430	11.7	113	9.5	162	13.6	1.4^ns^
Fasting	35	1.3	4	0.4	5	0.5	6.3**
**Females**	4273	53.1	1536	56	1530	56.2	-
Binge eating	491	16.8	60	6.9	50	5.6	95.7***
CB - purging:							
Self-induced vomiting	55	1.3	27	1.8	10	0.6	2.3^ns^
Taking laxatives	9	0.2	7	0.5	4	0.3	0.3^ns^
Taking diuretics	12	0.3	6	0.4	3	0.2	0.1^ns^
CB - non-purging:							
Taking diet pills	20	0.5	23	1.5	31	2.1	30.1***
Excessive training	602	14.3	178	11.7	143	9.4	25.9***
Fasting	25	0.8	2	0.2	1	0.1	8.1**

*p < .05, **p < .01, ***p < .001, *n *number, *ns *non significant, *df *degrees of freedom, *CB *Compensatory behaviours, *T1, T2, and T3 *time points 1, 2 and 3, respectively

^1^Prevalence is assigned only to those individuals who had behaviours at least once per week.

^a^Significant gender differences at T1 (*p *< .05)

^b^Significant gender differences at T2 (*p *< .05)

^c^Significant gender differences at T3 (*p *< .05)

**Figure 1:**
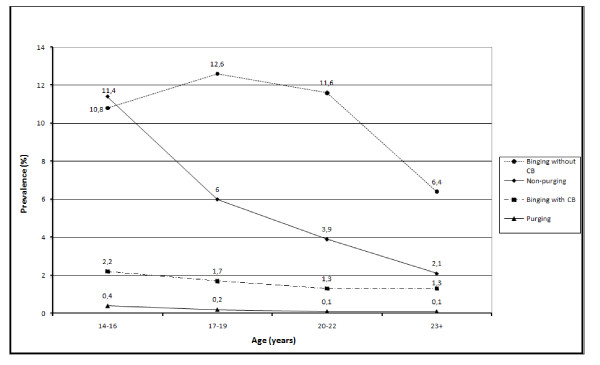
**Prevalence for four groups of binge eating and compensatory behaviours (CB) from adolescence to young adulthood for males**.

**Figure 2:**
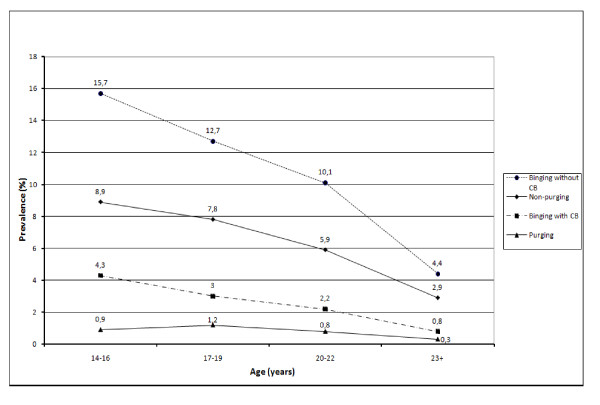
**Prevalence for four groups of binge eating and compensatory behaviours (CB) from adolescence to young adulthood for females**.

Figures 1 and 2 illustrate changes for males and females, respectively, in the prevalence of binge eating and CB for our four subclinical ED subgroups from adolescence to young adulthood. To ensure an adequate sample size in each age group and to describe changes in prevalence at specific ages, we categorised the age of participants into 14-16, 17-19, 20-22, and 23 years and above. Chi square tests showed significant trends for the purging and non-purging CB groups for both females (purging: *χ***^2 ^**(1) = 5.8, p = 0.016; non-purging: *χ***^2 ^**(1) = 55.2, *p *< 0.001) and males (purging: *χ***^2 ^**(1) = 4.2, *p *= 0.040; non-purging: *χ***^2 ^**(1) = 101.8, *p *< 0.001), indicating a decrease in the prevalence of these behaviours from age 14-16 to age 23 and over for both genders. For the binging without CB and binging-CB groups, we found significant decreases in prevalence from age 14-16 to age 23 and over for females (binging without CB: *χ***^2 ^**(1) = 34.9, *p *< 0.001; binging-CB: *χ***^2 ^**(1) = 12.4, *p *< 0.001) but no significant changes for males (*p *> 0.05).

To further address the effects of age and gender, logistic random intercept models were estimated, with age and gender as independent variables. Table 2 presents univariate regression estimates of age and gender effects for each of the four groups. Due to a high collinearity between age and time, we did not control for the time of measurement effects. These analyses controlled for dependency between repeated observations. To run such longitudinal models, we limited the analyses to participants who had responded to at least two of the three questionnaires from T1 to T3 (n = 3053). The univariate models show that age had a significant effect on each of the four symptom variables; risks of binge eating and CB declined with age. A significant gender difference was found only for purging, with females at higher risk. We also tested the effect of gender × age interactions in multiple regression models and found that the interaction term was significant only for the group displaying symptoms of binging without CB (OR = 0.90, 95% CI = 0.83-0.97, *p *= 0.009); binge eating declined more markedly for females than for males.

**Table 2:** Logistic random intercept model results for age and gender effects on binge eating and compensatory behaviours groups

Variables	Binging without CB	Binging with CB	Purging	Non-purging
	
	OR (95% CI)	OR (95% CI)	OR (95% CI)	OR (95% CI)
Age	0.89(0.86-0.93)***	0.91(0.85-0.98)*	0.91(0.84-0.98)*	0.86(0.84-0.89)***
Gender	1.01(0.73-1.41)	1.24(0.69-2.21)	3.51(1.35-9.11)*	1.17(0.88-1.55)

*p < .05, **p < .01, ***p < .001; *OR *odds ratio, *CI *confidence interval, *CB *compensatory behaviours

We then conducted further analyses (ANCOVA) to examine whether the four groups differed on measures of general eating and psychosocial problems, using age and gender as covariates. In addition, we compared the findings from these analyses with corresponding findings from participants who reported no symptoms of either binge eating or CB (NoS). Due to the comparatively small sample sizes in the purging and binging-CB groups at T2 and T3, the analyses were based on data from T1 only.

As Table 3 shows, all scores for measures of general eating and psychosocial problems, with the exception of overprotection and social support, differed significantly among the groups. The *post hoc *values shown in Table 4 indicate that the purging CB group had significantly lower levels of appearance satisfaction and parental care, more anxiety and depressive symptoms and higher scores on the EAT-12 and BITE-30 scales than all other groups. Purgers also reported more alcohol consumption than the binging without CB group and those with no symptoms for binging or CB, and higher scores on instability of self-concept and loneliness when compared with the binging without CB, non-purging and no-symptom groups. The binging-CB and non-purging CB groups had comparable scores on most of general eating and psychosocial measures; we found no significant differences between these two groups on any of the variables. Both groups reported significantly lower levels of appearance satisfaction, more anxiety and depressive symptoms, higher scores on the EAT-12 and BITE-30 scales and a higher score for instability of self-concept than the binging without CB and no-symptom groups. In contrast, when compared to the other three groups, the binging without CB group showed a significantly higher level of appearance satisfaction, and lower scores for anxiety and depressive symptoms, instability of self-concept and the EAT-12 and BITE-30 scales. Furthermore, individuals in the binging without CB group did not differ significantly from participants without ED symptoms on any of the variables except alcohol consumption, on which they scored significantly higher.

**Table 3:** ANCOVA results for rating groups of binge eating and compensatory behaviours on measures of general eating and psychosocial problems 1

Variables	Binge eating without CB (n = 726)	Binge eating with CB (n = 133)	Purging CB (n = 56)	Non-Purging CB (n = 624)	NoS (n = 4016)	ANCOVA
	
	Mean (s.d.)	Mean (s.d.)	Mean (s.d.)	Mean (s.d.)	Mean (s.d.)	F (d.f. 4)
BMI	21.40(4.80)	21.78(2.83)	21.86(3.04)	22.03(2.77)	21.38(4.81)	5.26**
Appearance satisfaction	3.47(0.64)	3.29(0.75)	2.82(0.88)	3.37(0.69)	3.52(0.64)	16.98***
Anxiety symptoms	1.46(0.49)	1.64(0.58)	1.98(0.73)	1.53(0.51)	1.45(0.46)	14.29***
Depressive symptoms	1.78(0.59)	2.02(0.74)	2.36(0.74)	1.85(0.64)	1.74(0.59)	16.75***
Self-worth	2.56(0.33)	2.48(0.38)	2.54(0.47)	2.54(0.34)	2.57(0.32)	2.52*
Alcohol consumption	2.67(1.64)	2.77(1.63)	3.40(1.58)	2.86(1.63)	2.79(1.66)	5.60**
EAT-12	1.55(0.35)	1.88(0.44)	2.43(0.63)	1.85(0.41)	1.54(0.36)	184.67***
BITE-30	1.49(0.39)	1.65(0.52)	2.17(0.59)	1.71(0.43)	1.50(0.41)	61.79***
Relationship to parents:						
Overprotection	2.03(0.58)	2.01(0.60)	2.09(0.69)	2.04(0.59)	2.01(0.57)	0.41^ns^
Care	3.09(0.56)	3.07(0.58)	2.72(0.67)	3.07(0.58)	3.08(0.57)	5.69**
Instability of self-concept	2.48(0.73)	2.69(0.74)	2.91(0.70)	2.65(0.70)	2.43(0.72)	16.06***
Social support	3.48(0.46)	3.49(0.51)	3.41(0.52)	3.47(0.48)	3.46(0.46)	0.56^ns^
Loneliness	1.81(0.56)	1.91(0.58)	2.17(0.69)	1.84(0.56)	1.83(0.56)	4.52*
Age (not controlled for covariates)	17.12(1.93)	16.91(1.91)	17.26(2.06)	16.92(2.01)	17.16(1.92)	2.63*

*p < .05, **p < .01, ***p < .001; *ns *non-significant, *SD *standard deviation, *df *degree of freedom, *BMI *body mass index, *EAT-12 *Eating Attitude Test, *BITE *Bulimic Investigatory Test, Edinburgh; *BMI *body mass index, *EAT-12 *Eating Attitude Test, *CB *compensatory behaviours; NoS - individuals who did not report either binge eating or compensatory behaviours

ANCOVA - Analysis of Covariance with age and gender as covariates

^1^Results are based on data from the first time point only (T1: 14-22 years of age)

**Table 4:** Tukey-Kramer *post hoc *comparisons of the groups of binge eating and compensatory behaviours1

Variables	**BEO v. BE**-**CB**	**BEO v. P**-**CB**	**BEO vs. NP**-**CB**	BEO v. NoS	**BE**-**CB v. P**-**CB**	**BE**-**CB v. NP**-**CB**	**BE**-**CB v. NoS**	**P**-**CB v. NP**-**CB**	**P**-**CB v. NoS**	**NP**-**CB v. NoS**
BMI	1.81	0.90	5.67**	2.48	0.33	1.41	0.80	1.39	0.15	4.96**
Appearance satisfaction	4.68**	11.85**	5.57**	1.54	7.31**	1.54	5.73**	9.59**	12.75**	8.77**
Anxiety symptoms	5.48**	12.15**	4.02*	0.53	7.18**	3.20	6.12**	10.51**	12.75**	5.76**
Depressive symptoms	6.65**	12.19**	5.26**	0.40	6.40**	3.67	6.92**	10.06**	12.77**	6.41**
Self-worth	4.26*	1.25	2.29	0.73	1.56	2.95	4.91**	0.31	1.53	3.72
Alcohol consumption	1.63	5.27**	4.89**	4.87**	3.52	1.11	0.46	3.29	3.93*	1.59
EAT-12	16.07**	28.53**	25.33**	0.39	14.65**	1.79	17.07**	18.29**	29.41**	32.66**
BITE-30	6.26**	19.94**	17.14**	3.07	13.31**	3.35	5.32**	13.06**	19.67**	19.31**
Relationship to parents:										
Overprotection	0.93	1.73	1.22	0.08	2.05	1.57	1.02	1.24	1.76	1.48
Care	0.16	6.03**	0.22	0.37	5.23**	0.28	0.32	5.89**	6.11**	0.05
Instability of self-concept	4.49*	7.20**	6.36**	2.87	3.40	0.97	5.96**	4.65**	8.25**	10.70**
Social support	0.99	0.44	0.88	0.62	0.99	0.51	1.32	0.78	0.27	1.72
Loneliness	2.43	5.41**	1.68	2.16	3.12	1.47	1.68	4.71**	4.95**	0.19

*Significant differences among groups at *p *< .05

**Significant differences between groups at *p *< .01

^1^Tukey-Kramer test values were derived from the analysis of covariance in Table 3

*BMI *body mass index, *EAT-12 *Eating Attitude Test, *BITE *Bulimic Investigatory Test, Edinburgh, *BEO *binge eating only (without CB), *BE-CB *binge eating combined with any of compensatory behaviour, *P-CB *purging behaviours, *NP-CB *non-purging behaviours; NoS - participants who did not report any binge eating and or behaviours

## Discussion

The main findings in this population-based longitudinal study are that the subclinical groups of individuals with different combinations of ED symptoms displayed distinct age- and gender-related trends. Females showed a significantly higher risk than males for purging behaviours. The prevalence of binge eating and CB in females and CB in males gradually declined with the transition from adolescence to young adulthood. Furthermore, individuals with subclinical forms of ED differed significantly on measures of general eating and psychosocial problems. Specifically, in terms of our psychosocial measures, purging emerged as the most serious type of behaviour when compared with binging without CB, binging-CB and non-purging CB. On the other hand, individuals in the binging without CB group were the least disturbed and in most aspects comparable to the reference group, i.e. those without inappropriate eating behaviours.

To our knowledge, this is the first population-based study documenting the relationship between distinct types of ED behaviour (binging without CB, binging-CB, purging and non-purging) and different measures of general eating and psychosocial problems. The study shows a particularly high symptom load in individuals who purged, since those who purged, largely by self-induced vomiting, had significantly higher levels of appearance dissatisfaction, anxiety and depressive symptoms, alcohol consumption, self-concept instability and loneliness. A national study of high school students in the USA also reported vomiting for weight control as the most clinically significant behaviour and one that may be a particularly deleterious component of ED [34]. In this respect, individuals engaging in purging behaviours should probably be targeted as a high-risk group.

In contrast, Mond and colleagues reported that the combination of routine binge eating and CB was associated with higher ED psychopathology and impairment in mental and physical functioning than was the occurrence of CB alone, regardless of the type, i.e. purging or non-purging [8]. These divergent findings may be the result of different definitions for binge eating and different scales for measuring psychopathology. We defined binge eating only in terms of the amount of food consumed. Mond et al., on the other hand, integrated loss of control in their definition, and this could add an important dimension to the relationship between binge eating and psychological disturbance. As well, Mond et al. used the Eating Disorder Examination Questionnaire and the Short-Form Disability Scale, whereas we used a wider range of eating and psychosocial measurements, providing in-depth information about the differences among the groups. In general, both community- and clinical-based studies indicate that individuals with purging subtype of bulimia nervosa have more severe psychopathology than those with non-purging bulimia nervosa and binge eating disorder, thereby demonstrating that purging behaviors may increase the severity level of psychopathology among those with the full-threshold ED [35,36]. These studies support our finding that purging behaviors are related to particularly serious levels of psychosocial problems. Due to few participants with a combination of binging and purging in our study, we could not investigate how individuals with purging only symptoms differed from those with a combination of purging and binging. More research would help to clarify distinctions between these groups, which, in turn, could have important implications for the future classification of subclinical ED.

To an extent, findings from this study parallel those of earlier community-based studies [16,37]. The prevalence of binge eating and several CB behaviours appears to be higher among females than males. The gender difference in the prevalence of symptoms was especially notable for purging, with females at a significantly higher risk. A possible explanation is that females have a stronger desire to lose weight and a higher drive for thinness than males, leading them to engage in more purging behaviours [16,37]. Alternatively, males may be more uncomfortable reporting purging behaviours than females [38].

In both genders, the age-related decline in prevalence rates for binge eating and CB may be partially explainable by roles associated with the transition to adulthood, specifically partnering and motherhood (for females) [17,39]. However, it may be possible that psychological problems which in many cases are associated with disordered eating may not be reduced to a comparable degree as disordered eating symptoms in the transition to adulthood. For instance, even though Patton et al. reported most part of adolescent eating disorders to be limited to the teens, co-morbid conditions, such as depression, anxiety and binge drinking persisted to a much larger degree into adulthood [14]. Such findings may indicate that the decline of symptoms of disordered eating not necessarily is related to symptom alleviation for other mental health problems.

Moreover, even though we saw this declining trend for both males and females, binge eating among females stands out in terms of the magnitude of the reduction over age. This may be related to a greater perception on the part of females that binge eating is a problematic behaviour [40]. Along the same line of thought, males report a more positive affect (feeling happy) than females after binging [41,42].

Overall, the prevalence rates of symptoms in this study are lower when compared with the rates from two studies of US college men and women [43,44]. They are, however, similar to rates found in two community-based studies conducted in Australia and the USA, respectively [5,8]. Nonetheless, factors such as the use of different assessment measures, lack of standard definitions for syndromes, differences in methodological approaches and characteristics of sample populations may limit the comparability of the findings.

This study has a number of limitations that warrant consideration. First, the BITE does not specify a time frame for the occurrence of symptoms, thereby providing somewhat limited information about the duration of a respondent's ED. Second, binge eating determined solely by the amount of food consumed may be a somewhat limited indicator for binge eating psychopathology. The inclusion of additional BITE items such as feeling distress after binging, binging alone, and urge to binge or loss of control might better distinguish binging from normal eating. This matter bears further investigation. Third, even though it has been argued that full-syndrome ED may not differ qualitatively from sub-threshold levels [45,46], it remains to be seen whether the findings from this study will be supported in studies using diagnostic categories of ED and in those using diagnostic interviews. Fourth, age-related differences in prevalence rates of ED behaviours may reflect time of measurement effects and differences in exposure to risk factors rather than effects associated with growing into adulthood. Other studies suggest that a wide range of ED behaviours remain relatively stable over time, supporting the negligible effect of time on the trend of prevalence [5,47]. Furthermore, separating the effect of age from period and cohort through statistical model estimation has proven problematic and has led to incorrect conclusions [48]. Fifth, limited statistical power resulting from the small sample sizes should be considered. More specifically, because the number of participants in the purging CB and binging-CB groups was rather small, potential differences among the groups might not have been detected. Sixth, to obtain more differentiated information, the binging-CB group should ideally have been divided into those who binged and purged and those who binged and engaged in non-purging CB. Unfortunately, the small number of participants reporting both binging and purging behaviours precluded such analyses. Finally, we only followed about 25% of the representative sample at T0. Even though most of the attrition was planned, and that attrition analyses showed no significant differences between those who dropped out and those who completed the study, the large proportion of drop out at the follow-up could be a source of bias.

## Conclusion

Our findings suggest that purging behaviours are the most severe and gender-bound subclinical forms of ED, i.e. behaviours indicating high-risk individuals. A substantial proportion of adolescents report binge eating and inappropriate weight control methods; the risk declines to some extent with the transition to adulthood. Early preventive interventions of such inappropriate eating behaviours have the potential to decrease the likelihood of progression to full-threshold ED. The differences found among the groups in this study may be useful in the discussion concerning a reformulation of clinical forms of ED in the Diagnostic and Statistical Manual of Mental Disorders-V, particularly around the necessity to capture diversities in the EDNOS category. It is important to explore more closely whether psychosocial disturbances cause or mediate the observed difference among the ED groups. Moreover, future studies should be aimed at finding factors that are potent in distinguishing individuals who experience disordered eating symptoms which are limited to the adolescent years from those who suffer from longer lasting symptoms which persist into adulthood. Research is as well needed examining in which way psychosocial correlates of disordered eating that were found in the present study are causally related to different types of eating disordered symptoms. Such studies would provide valuable information for prevention and intervention efforts in the field of disordered eating.

## Competing interests

The authors declare that they have no competing interests.

## Authors' contributions

DSA analysed the data, interpreted the results and drafted the manuscript. All authors participated in the write-up, contributed to the interpretation of the study results and approved the final version of the manuscript submitted for publication.

## Pre-publication history

The pre-publication history for this paper can be accessed here:

http://www.biomedcentral.com/1471-2458/12/32/prepub
